# Characterizing macrophage diversity in colorectal malignancies through single-cell genomics

**DOI:** 10.3389/fimmu.2025.1526668

**Published:** 2025-03-21

**Authors:** Tingshuo Zhao, Yinyi Luo, Yuanjie Sun, Zhigang Wei

**Affiliations:** ^1^ First Clinical Medical College, Shanxi Medical University, Tai Yuan, China; ^2^ Department of Hepatobiliary and Pancreatic Surgery, The First Hospital of Shanxi Medical University, Tai Yuan, China

**Keywords:** colorectal cancer, tumor-associated macrophages, single-cell RNA sequencing, tumor microenvironment, M2-macrophage

## Abstract

Colorectal cancer (CRC) is one of the most common malignant tumors of the digestive tract, with increasing incidence and mortality rates, posing a significant burden on human health. Its progression relies on various mechanisms, among which the tumor microenvironment and tumor-associated macrophages (TAMs) have garnered increasing attention. Macrophage infiltration in various solid tumors is associated with poor prognosis and is linked to chemotherapy resistance in many cancers. These significant biological behaviors depend on the heterogeneity of macrophages. Tumor-promoting TAMs comprise subpopulations characterized by distinct markers and unique transcriptional profiles, rendering them potential targets for anticancer therapies through either depletion or reprogramming from a pro-tumoral to an anti-tumoral state. Single-cell RNA sequencing technology has significantly enhanced our research resolution, breaking the traditional simplistic definitions of macrophage subtypes and deepening our understanding of the diversity within TAMs. However, a unified elucidation of the nomenclature and molecular characteristics associated with this diversity remains lacking. In this review, we assess the application of conventional macrophage polarization subtypes in colorectal malignancies and explore several unique subtypes defined from a single-cell omics perspective in recent years, categorizing them based on their potential functions.

## Graphical Abstract

The distinctions between classical subtypes and new variants.

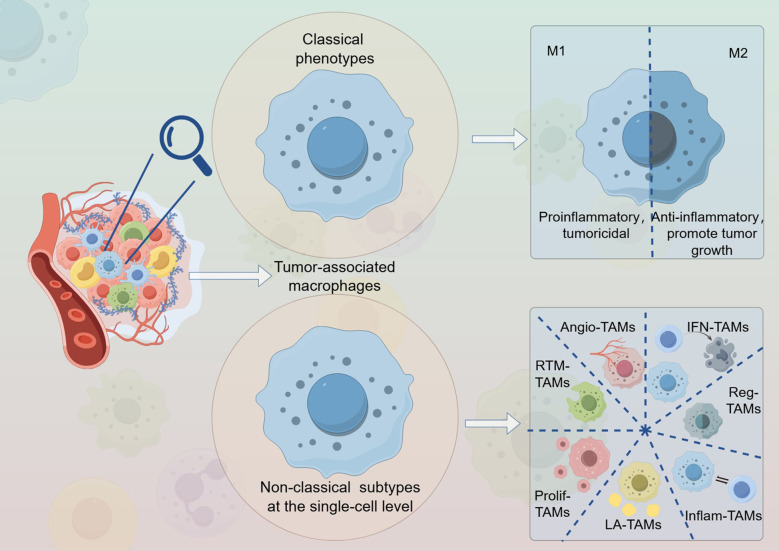

## 1 Introduction

Colorectal cancer (CRC) is one of the most prevalent malignant tumors of the digestive tract and the second leading cause of cancer-related mortality worldwide (1). Both its incidence and mortality rates are rising annually (2), In 2020, it was estimated that there were over 1.9 million new cases of CRC and approximately 930,000 deaths attributed to the disease. The burden of CRC is projected to rise significantly, with an anticipated increase to 3.2 million new cases and 1.6 million deaths by the year 2040. Most of these cases are expected to occur in countries classified as having a high or very high Human Development Index (3), posing a significant challenge to public health. Early-stage CRC is typically managed with surgical resection combined with chemotherapy. However, due to its high malignancy and substantial risks of metastasis and recurrence, traditional treatment outcomes are often unsatisfactory (4). Identifying new therapeutic targets is, therefore, crucial for improving the prognosis of patients with CRC.

Current research has identified that the mechanisms underlying colorectal cancer (CRC) are diverse, including factors such as gut microbiota (5, 6) and epigenetic alterations (7, 8). However, the complex molecular mechanisms remain to be further elucidated. There is substantial evidence indicating that immunoediting plays a significant role in tumors (9, 10). In CRC patients, immune balance is disrupted (11), and the interactions between tumor cells and the tumor microenvironment (TME) facilitate further tumor progression (12, 13). Macrophages, crucial immune components of the TME, play a significant role in recognizing and phagocytosing pathogens as well as necrotic cells, thereby contributing to immune regulation and maintaining tissue homeostasis (14, 15). Under various physiological and pathological conditions, macrophages can differentiate into distinct phenotypes based on their gene expression profiles. These diverse subpopulations coordinate tumor progression, metastasis, and invasion by secreting specific cytokines and chemokines (16). Macrophage heterogeneity offers new insights into the diagnosis, treatment, and prognosis of CRC.

Tumor-associated macrophages (TAMs) are among the most abundant immune cells in tumor tissues. Traditionally, macrophages have been classified into two subtypes: M1 (classically activated or proinflammatory) and M2 (alternatively activated or anti-inflammatory) (17). The M1 subtype is characterized by tumoricidal properties, while the M2 macrophages exhibit anti-inflammatory characteristics that can indirectly promote tumor growth (18, 19). Although this binary classification has been widely utilized in the past, technological advancements have expanded our understanding of the complex state of the TME. Consequently, this dichotomy fails to accurately capture the diversity of macrophage phenotypes present *in vivo*, particularly within complex TME contexts (20–22). Furthermore, the gene expression profiles associated with these two phenotypes are not entirely oppositional (23). With advances in single-cell RNA sequencing (scRNA-seq), researchers can now identify cellular subpopulations with greater precision. This technology enables the discovery of unique macrophage subpopulations by capturing transcriptomic variations in macrophages under different pathological conditions and disease stages, thereby distinguishing various functional subsets (24, 25). Additionally, the integration of scRNA-seq with spatial transcriptomics has enhanced the ability to detect finer details within heterogeneous cell populations (26, 27). This article focuses on non-classical classifications of TAMs identified through single-cell omics research on colorectal malignancies and explores their potential roles in tumorigenesis and development.

## 2 Overview of tumor-associated macrophages

Macrophages are phagocytic cells widely distributed in various human tissues and organs. They play crucial roles in recognizing, phagocytosing, and degrading apoptotic cells, tissue debris, and pathogens, thereby contributing significantly to immune responses. It is currently understood that macrophages within tissues have two primary sources (28): during prenatal development, they originate from yolk sac progenitors or hematopoietic stem cells. These progenitor cells retain proliferative potential and can undergo self-renewal. During organ development, they differentiate into specific tissue subpopulations (29, 30), such as microglia in the central nervous system and Kupffer cells in the liver. This differentiation establishes stable communication and connections with specialized tissue cells (31, 32). In contrast, adult macrophages arise from infiltrating monocytes and are involved in a wide range of physiological and pathological processes within the body. They play central roles in various processes, including inflammation regulation and tissue repair (33). Both types of macrophages can coexist within specific tissues, and their respective abundance reflects the characteristics and history of these tissues (34).

Macrophages are highly plastic cells (35) and the generation of TAM is multifaceted. Chemokines and cytokines, such as monocyte chemoattractant protein-1 (MCP-1) and colony-stimulating factor 1 (CSF1), along with complement cascade products, are the primary determinants of the recruitment and localization of macrophages within tumors. Under the influence of these factors, peripheral blood monocytes are locally recruited and differentiate into macrophages, forming TAMs alongside resident tissue macrophages (36–39). Proinflammatory cytokines produced by tumor cells contribute to the recruitment of immune cells and promote macrophage polarization during the early stages of carcinogenesis. Proper activation of macrophages can exert certain anti-tumor effects by enhancing phagocytosis and cytotoxicity against tumor cells. Additionally, macrophages can induce extracellular killing of tumor cells through antibody-dependent cellular cytotoxicity (ADCC) (40).However, it is notable that macrophages in the TME do not appear to exert protective effects; rather, a substantial body of research indicates that they seem to promote tumor progression in the vast majority of circumstances (41, 42).

Based on the functions of TAM, two activation phenotypes, M1 and M2, have been studied extensively. M1 macrophages respond to various stimuli, such as bacteria, interferon-γ (IFN-γ), lipopolysaccharide (LPS), and tumor necrosis factor-α (TNF-α). They exhibit enhanced antigen presentation and complement-mediated phagocytosis, contributing to the inflammatory response (43, 44). Additionally, M1 macrophages activate or recruit adaptive immune cells and are capable of phagocytosing and killing tumor cell (45). In this stage, M1 macrophages specifically produce cytotoxic substances such as nitric oxide (NO) and reactive oxygen species (ROS), which can damage tumor cells, participate in phagocytosis, and release proinflammatory cytokines. This process further stimulates anti-tumor immunity (46–48). Moreover, tumor cells induce the polarization of macrophages into M2-type TAMs through various mechanisms, including cytokine production, metabolic abnormalities, and hypoxia (49–54). M2 macrophages can be categorized into the subtypes M2a, M2b, M2c, and M2d based on their activation factors (55). Subtypes M2a and M2b primarily exert immunoregulatory functions by supporting T helper cell 2 (Th2)-mediated responses, whereas subtypes M2c and M2d are involved in immune suppression and tissue remodeling (56). TAMs of the M2 phenotype predominantly inhibit tumor immune responses by secreting immunosuppressive factors, thereby disrupting the immune barrier and leading to an imbalance in defense mechanisms. Additionally, they secrete growth factors such as transforming growth factor (TGF) and vascular endothelial growth factor (VEGF), which promote tumor proliferation and extracellular matrix (ECM) remodeling. M2-type TAMs play a significant role in promoting angiogenesis, tissue repair, and tumor progression (47).

In the TME, TAMs typically exhibit an M2 phenotype, which facilitates tumor malignancy either by directly enhancing malignant biological behaviors or by engaging in crosstalk with immune cells present in the TME to promote tumor progression (57). However, increasing evidence suggests that the polarization state of macrophages is far more complex than the simplistic M1/M2 dichotomy.

The elevation of TAMs is associated with poor prognoses in various cancers (16, 58, 59). M2 TAMs can further promote tumor progression (14, 57, 60, 61). This correlation primarily arises from their tumor-promoting characteristics, which include facilitation of angiogenesis, immune suppression, and promotion of cancer cell dissemination. TAMs regulate angiogenesis in the TME by releasing pro-angiogenic factors like vascular endothelial growth factor (VEGF), basic fibroblast growth factor (bFGF), chemokines, and antiangiogenic substances such as thrombospondin-1 (TSP-1). Consequently, TAMs play a complex role in vascular formation (42, 62–64). Moreover, TAMs promote tumor progression by suppressing immune responses (65). They achieve this by expressing T cell immune checkpoint ligands that attract regulatory T cells (Tregs) and by producing immunosuppressive molecules such as transforming growth factor (TGF) and interleukin-10 (IL-10), which impair the function of cytotoxic T cells (CTLs) and natural killer (NK) cells (66). TAMs inhibit the recruitment and function of T-cells via cytokines, surface immune checkpoint ligands, and exosomes. They play a crucial role in regulating programmed death receptor-1 (PD-1) and its ligand PD-L1-mediated immunosuppression (67). Additionally, TAMs can suppress T cells infiltration into tumor tissues, thereby promoting tumor growth (39). Furthermore, TAMs secrete matrix metalloproteinase-9 (MMP-9), which induces epithelial-mesenchymal transition (EMT) via the transcription factor Snail, enhancing tumor invasiveness (68). Other studies have shown that TAMs co-localize with tumor cells undergoing EMT (69); they also produce chemokines that facilitate distant tumor metastasis (70). The recruitment of cancer-associated fibroblasts (CAFs) by TAMs is associated with extracellular matrix remodeling, promoting tumor invasion (71, 72). In summary, TAMs exhibit dynamic roles throughout tumor development and engage in complex interactions within the TME.

## 3 The constraints of classical typological frameworks

TAMs exhibit significant heterogeneity not only among different patients with cancer but also within various malignant lesions of the same patient and even within specific tumor sites. The intratumoral heterogeneity of macrophages may pose challenges for treatment, yet it also underscores the complexity of their functions (72–77). Due to its low resolution, traditional bulk RNA sequencing fails to accurately represent subtle variations in cell populations within the TME (78, 79). In contrast, single-cell RNA sequencing (scRNA-seq) allows for a comprehensive and detailed characterization of cellular diversity and heterogeneity at the single-cell level (78), thereby providing a more nuanced perspective on the complexity of TAMs. The traditional M1/M2 classification of macrophages faces certain challenges, primarily for the following reasons: 1. Diversity of Macrophages: Macrophages exhibit a high degree of heterogeneity *in vivo*, and their functions and phenotypes are influenced by various factors and complex interactions with the microenvironment (80–82). The relatively simple polarization into M1 and M2 macrophages may not fully encompass the intricate spectrum of macrophage subtypes present in the body. For instance, studies have identified other macrophage subtypes, such as SPP1+ TAMs (83–85) and FOLR2+ tissue-resident macrophages (TRMs) (86, 87), which include populations that are significant for prognosis in patients with cancer but do not conform to the classical M1/M2 polarization paradigm. 2. Inaccuracy of Single Markers: The classification of M1 and M2 macrophages often relies on specific markers such as CD68, CD163, and CXCL10. However, the expression of these markers may overlap between different subtypes, leading to inaccuracies in classification; thus, it is essential not to consider these markers in isolation (88–90). Furthermore, variations in the markers used and the experimental conditions across different studies may further impact the classification accuracy. 3. Misunderstanding of the Dynamic Balance: The simplistic classification of macrophages into M1 and M2 types may lead to misconceptions regarding the dynamic balance of macrophages *in vivo*. Macrophages undergo phenotypic transitions from one subtype to another in response to various stimuli. This dynamic change is crucial for inflammatory responses and immune regulation; however, a binary classification approach fails to accurately capture the true state of macrophages (91, 92). Research has evolved from straightforward and convenient classical M1 and M2 models to more complex models that reflect the diverse functional spectra of macrophages (93, 94). This increasing level of detail necessitates the distinction between the subtypes and functions of TAMs from the perspective of single-cell sequencing.

## 4 Identification of novel non-classical macrophage subtypes at the single-cell level in malignant colorectal tumors

Ma et al. (94) summarized seven subgroups of TAMs identified in various pan-cancer single-cell studies (23, 94) and classified them based on their functions, signatures, and enriched pathways. These subgroups include interferon-primed TAMs (IFN-TAMs), immune regulatory TAMs (Reg-TAMs), inflammatory cytokine-enriched TAMs (Inflam-TAMs), lipid-associated TAMs (LA-TAMs), pro-angiogenic TAMs (Angio-TAMs), RTM-like TAMs (RTM-TAMs), and proliferating TAMs (Prolif-TAMs). Notably, functional overlaps and shared characteristics are present among these seven subtypes. In subsequent sections, we outline the unique phenotypes and functions of specific TAM subpopulations discovered in single-cell research on CRC. Meanwhile, more concise information can be more readily accessed in **Table 1**.

**Table 1:** Non-classical macrophage subtypes at the single-cell level in malignant colorectal tumors.

Phenotype	Categorization	Features/Functions	Refs
SPP1+	Pro-angiogenic TAMs	Enhancing the expression of CCL18 and FTL, which exhibit pro-angiogenic functions.	(101)
SPP1+	Pro-angiogenic TAMs	Exhibiting a high angiogenesis score.	(106)
SPP1+	Pro-angiogenic TAMs	Highly associated with FAP+ fibroblasts and may play a role in ECM remodeling.	(107)
SPP1+	Immune regulatory TAMs	The pathways of antigen processing and presentation and T-cell co-stimulation are enriched in SPP1+TAMs.Enrichment of the Wnt signaling pathway. High expression of MHC class II genes such as HLA-DRB5, HLA-DQA1, and HLA-DQB1.	(138)
SPP1+	Inflammatory cytokine-enriched TAMs	Most of these ligands associated with lymphocyte exhaustion markers are secreted by myeloid cells, represented primarily by SPP1+ macrophages.	(123)
SPP1+	Inflammatory cytokine-enriched TAMs	The enrichment of the Wnt signaling pathway, along with the KLF, specifically KLF14 and KLF16, is observed.	(138)
SPP1+	Interferon-primed TAMs	Immunosuppression and tumor promotion	(84)
SPP1+	Interferon-primed TAMs	High expression of CTLA-4 induces T cell exhaustion.	(123)
SPP1+	Interferon-primed TAMs	Highly expression of CCL20 promotes the migration and proliferation of cancer cells, as well as remodels the tumor microenvironment to accelerate cancer progression.	(101)
SPP1+	Interferon-primed TAMs	The high expression of NAMPT is associated with the immunosuppressive functions of M2 macrophages.	(129)
MS4A4A+	Lipid-associated TAMs	Highly expressed characteristic genes such as C1QA and APOE.The expression of M2 macrophage marker genes in MS4A4A+ TAMs within tumor samples is significantly higher than that in normal tissues, indicating an immunosuppressive role.	(145)
MRC1+CCL18+	Lipid-associated TAMs	A group of immunosuppressive cells exhibits significant enrichment in metabolic pathways.	(148)
MMP12+	Pro-angiogenic TAMs	Enriched in biological processes such as cell migration and angiogenesis. Differentiated into a lineage with enhanced angiogenic capabilities through pseudotime analysis.	(111)
MMP12+	Interferon-primed TAMs	The interaction between SPP1-CD44 ligands on T cells and B cells is enhanced, thereby inhibiting T cell activation. The interaction between CD86-CTLA4 ligand-receptor pairs on T cells and B cells is also strengthened.	(111)
MK67+	Proliferating TAMs	The expression of MKI67 may be associated with its proliferative function.	(106)
GPNMB+	Pro-angiogenic TAMs	The functionality is enriched in angiogenesis and is spatially closer to the tumor margin in spatial transcriptomics.	(98)
C1QC+	RTM-like TAMs	Exhibiting phagocytic and antigen-presenting functions, while also playing a potential role in the recruitment or activation of T cells. It is closely associated with certain specific RTMs and shares some similarities in functional phenotype.	(85)
C1QC +	Pro-angiogenic TAMs	Interaction with MFAP5+ fibroblasts in tumor tissue and facilitating tumor progression.	(115)
APOE+CTSZ+	Interferon-primed TAMs	High expression of immunosuppressive markers. The high expression of CXCL16 exacerbates immune suppression through its interaction with CXCR6 on Treg cells.	(135)

### 4.1 Pro-angiogenic TAMs

The characteristics of Angio-TAM are marked by the high expression of angiogenesis-related features, including vascular endothelial growth factor-A and SPP1, as well as other angiogenic factors. These growth factors promote angiogenesis in various cancers (95). The formation of new blood vessels plays a crucial role in tumor progression and metastasis, where the tumor vasculature regulates oxygen supply to support tumor growth and facilitates the dissemination of cancer cells (96). Additionally, this macrophage subset may be involved in the process of EMT, another significant process that contributes to metastasis. During EMT, epithelial cells lose their attachment to the basement membrane and intercellular junctions, transforming into mesenchymal cells characterized by enhanced migratory and invasive properties (97). Furthermore, complex interactions between this subset and cancer-associated fibroblasts have been observed. Therefore, Angio-TAM may exert its influence on tumor progression through multiple mechanisms.

A study (98) conducted single-cell sequencing on matched specimens from three patients, comparing the invasive margin (IM) of colorectal liver metastases (CLM) with adjacent normal areas (NA). The differentially expressed genes identified in TAMs compared to Kupffer cells included *GPNMB*, *TREM2*, *LGALS3*, and *FABP4*; these genes exhibited distinct expression profiles within TAMs. Glycoprotein Non-Metastatic B (GPNMB) is a heavily glycosylated transmembrane protein initially discovered in melanoma cells, osteoblasts, and dendritic cells. It has been proposed as a potential therapeutic target for CRC and is possibly associated with poor tumor prognosis (77, 99). Furthermore, increased GPNMB expression has been observed in macrophages exposed to tumor-conditioned medium *in vitro (* 100). The study found that GPNMB+ TAMs were primarily enriched in angiogenesis-related pathways. Spatial transcriptomic analysis revealed that these cells were localized closer to the tumor margins and were almost absent from the NA region, suggesting their potential roles in promoting angiogenesis.

In a study that integrated three datasets (101), CCL18 was enhanced in SPP1+ macrophages, which accelerated cancer progression by promoting angiogenesis (102, 103). The ferritin light chain (FTL) also possesses pro-angiogenic properties (104, 105), and its expression is upregulated in SPP1+ macrophages. A comprehensive investigation of the immune phenotypes associated with colorectal cancer liver metastases (106) identified three types of TAMs. Among them, SPP1+ TAMs were primarily enriched at the hepatic metastases. Subsequently, the clinical relevance of SPP1+ TAMs was explored. In the TCGA cohort, patients exhibiting higher levels of SPP1+ TAM characteristics were associated with poorer overall survival (OS) compared to untreated patients. Notably, the angiogenesis score for SPP1+ TAMs was the highest, corroborating previously established findings on their angiogenic function in CRC (85). A significant study focusing on FAP+ fibroblasts (107) identified tumor-specific macrophages that were highly associated with FAP+ fibroblasts, namely SPP1+ TAMs. These macrophages exhibit elevated expression levels of SPP1 and MARCO (Macrophage Receptor with Collagenous Structure, a scavenger receptor) (108, 109), and their proportion among myeloid cells in tumor samples is significantly higher than that in normal tissues. Subsequently, the study revealed a strong correlation between FAP+ fibroblasts and SPP1+ macrophages. Patients exhibiting high levels of both FAP+ fibroblasts and SPP1+ macrophages demonstrated the shortest progression-free survival period, suggesting the potential synergistic effects of these cell types in promoting tumor progression. Immunofluorescence labeling indicated that SPP1+ and FAP+ cells were in close proximity within CRC tissues, implying potential crosstalk between these two cell types. FAP+ fibroblasts and SPP1+ macrophages appear to form an interactive network that supports their maintenance and function. Both cell types may play critical roles in extracellular matrix (ECM) remodeling, possibly fostering the formation of collagen-rich areas within the TME (110).

A recent study (111). MMP12, a member of the matrix metalloproteinase (MMP) gene family secreted by macrophages (112), may serve as a marker of poor tumor prognosis (113, 114). The authors observed that the proportion of MMP12+ TAMs gradually increased across normal tissues, tumor tissues, and liver metastatic samples. Furthermore, MMP12-expressing macrophages exhibited high expression levels in various biological processes, including cell migration, lipid metabolism, angiogenesis, negative regulation of apoptosis, negative regulation of T-cell receptor signaling pathways, and negative regulation of T-cell proliferation. Using Monocle2 to construct the differentiation trajectory of MMP12+ macrophages revealed that, with the progression of pseudotime, the proportion of these cells increased in both tumor and liver metastatic samples. This differentiation led to two distinct branches; one branch was associated with enhanced angiogenic capacity.

In a single-cell study exploring MFAP5+ fibroblasts combined with spatial transcriptomics (115), MFAP5+ fibroblasts and macrophages were co-enriched in the fibroblast region, while certain adaptive immune cells, such as B and T cells, were notably absent from this area. TAMs expressing high levels of C1QC, C1QA, and C1QB were identified as C1QC+ TAMs. Further investigation revealed a significant positive correlation between the infiltration of MFAP5+ fibroblasts and C1QC+ macrophages. The signature scores for these cell types were calculated using two algorithms: ssGSEA (116) and AddModuleScore (117). These results indicated that MFAP5+ fibroblasts and C1QC+ macrophages were enriched within the fibroblast region. In normal tissues, there were few C1QC+ macrophages surrounding MFAP5+ fibroblasts, suggesting that crosstalk between these cell types occurred primarily within the tumor tissues. When visualizing the expression of ligands and receptors in the CSF/IL34/CSF1R signaling axis, it was noted that in normal tissues, MFAP5+ fibroblasts predominantly secreted CSF-1, which is recognized as a classic tumor-promoting factor that recruits macrophages to tumor sites and facilitates the polarization of TAMs (118), to interact with C1QC+ macrophages; however, in tumors, MFAP5+ fibroblasts exclusively released IL-34, another cytokine capable of binding to CSF1R on C1QC+ macrophages. IL-34 promotes the differentiation into the M2 phenotype across various cancers through its interaction with CSF1R while also facilitating the dysregulation of the TME toward a pro-tumorigenic state (119–122). These findings suggest that MFAP5+ fibroblasts regulate the phenotype of C1QC+ macrophages via the IL-34/CSF1R signaling pathway. In summary, complex interactions exist between fibroblasts and TAMs, leading to their polarization and functional transformations.

### 4.2 Interferon-primed TAMs

The IFN-TAM is a subtype that tends to exhibit immunosuppressive characteristics, characterized by high expression of IFN-regular genes. These macrophages’ primary functions include T cell exhaustion, immune suppression, inhibition of T cell activity, promotion of tumor cell proliferation, and recruitment of regulatory Treg.

A large-scale study (84) indicated that SPP1+ macrophages may exhibit immunosuppressive and tumor-promoting effects in patients with CRC, aligning with functions defined as IFN-TAMs. Japanese researchers (123) confirmed that cytotoxic T lymphocyte-associated antigen-4 (CTLA-4) is highly expressed in SPP1+ macrophages at the tumor invasion front, which are considered to be markers of exhaustion. This suggests that these macrophages deplete other immune cells at the invasive front, leading to T cells inactivity against tumor cells and T lymphocyte exhaustion (124, 125). A study integrating three datasets (101) revealed enhanced expression of CCL20 in SPP1+ macrophages, which is believed to facilitate cancer cell migration and proliferation, while remodeling the TME to accelerate cancer progression (126–128). Additionally, research conducted by Korean scholars (129) demonstrated elevated levels of nicotinamide phosphoribosyl transferase (NAMPT) expression in SPP1+ TAMs. This finding may be associated with the immunosuppressive function of M2-type macrophages, which contribute to the immunosuppressive microenvironment within CRC.

In the study by Fan et al. (111), Monocle2 was utilized to construct a differentiation trajectory of MMP12+ macrophages, revealing an additional branch characterized by enhanced TGF-β signaling and oxidative stress activity. In metastatic liver samples, MMP12 macrophages exhibit increased interactions with T and B cells through the SPP1-CD44 ligand (130). Macrophages express SPP1, which inhibits T cells activation via its interaction with CD44 (131), potentially promoting liver metastasis. Furthermore, the interaction between MMP12 macrophages and the CD86-CTLA4 ligand-receptor pair in T and B cells was significantly enhanced compared to that in normal samples; this interaction is critically important for tumor immunity (132–134).

Additionally, a study integrating two sets of CRC single-cell data focusing on metabolism (135) revealed that a subset of APOE + CTSZ + TAMs was highly enriched in the hypoxic group and exhibited elevated anti-inflammatory and M2-like scores. Differential gene expression analysis indicated that known immunosuppressive markers such as APOE, CTSZ, SEPPI, MRC1, and CD163 were significantly upregulated in the APOE + CTSZ + TAM group. Subsequently, the authors conducted receptor-ligand interaction analyses and discovered chemokine-receptor pairs between APOE + CTSZ + TAMs and Treg cells, including CXCL16-CXCR6. Notably, CXCL16 was highly expressed in APOE + CTSZ + TAMs, while CXCR6 was predominantly expressed in Treg cells. This suggests that APOE + CTSZ + TAMs may utilize the CXCL16 signaling pathway to recruit Tregs to tumor sites, thereby further exacerbating the immunosuppressive TME (136, 137).

### 4.3 Immune regulatory TAMs

The characteristic functions of Reg-TAM include T cell suppression, antigen presentation pathways, and immune checkpoints. In a study conducted by Che et al. (76), Reg-TAM was defined as exhibiting high expression of major histocompatibility complex (MHC) and co-stimulatory genes, which are indicative of immune activation and anti-tumor activity; however, it also shows elevated expression of immunosuppressive genes. This “double-agent” role in immune modulation suggests a complex interplay between anti-tumor and pro-tumor activities.

Reg-TAMs refer to immune regulatory TAMs, in a previous study (138), four macrophage subpopulations were identified, with C1QC+ and CD55+ macrophages preferentially enriched in adjacent tissues. SPP1+ and CXCL5+ macrophages, primarily enriched in tumor tissues, were designated as TAMs. Notably, SPP1+ TAMs exhibited both pro- and anti-inflammatory phenotypes. The authors conducted gene set variation analysis (GSVA) (139) and found that SPP1+ TAMs significantly enriched the pathways of “antigen processing and presentation” and “T-cell co-stimulation.” Furthermore, the authors observed that SPP1 + TAM exhibited enrichment in the Wnt signaling pathway, which may support tumor growth. Additionally, using the Monocle2 algorithm to reconstruct pseudo-time trajectories (140) revealed that SPP1+ and C1QC+ TAMs represent two distinct branches in the monocyte differentiation pathway. Consistent with the abundant antigen processing and presentation pathways observed in SPP1+ TAMs, there was elevated expression of MHC class II genes, such as HLA-DRB5, HLA-DQA1, and HLA-DQB1 (141)compared with the CXCL5+ TAM subpopulation. This further corroborates the complexity of the immune functions associated with this particular subpopulation. Based on this evidence, SPP1+ TAMs can be classified as immune regulatory TAMs.

### 4.4 Inflammatory cytokine-enriched TAMs

The primary characteristic of Inflam-TAMs is the expression of inflammatory cytokines, which may actively recruit and regulate immune cells in tumor-associated inflammatory responses, thereby promoting inflammation. Additionally, Inflam-TAMs may be involved in the Wnt signaling pathway as well as immune checkpoint responses.

One study indicated that, at the forefront of tumor invasion, two populations of tumor cells co-localized more frequently with SPP1+ macrophages than with other cell types (123). The authors analyzed the expression of lymphocyte exhaustion markers and various ligands associated with these markers such as PD-1/PD-L1. Most of these ligands are secreted by myeloid cells, represented primarily by SPP1+ macrophages, indicate that SPP1+ TAM may deplete other immune cells at the forefront of tumor invasion. To identify populations with lymphocyte recruitment capabilities, the authors analyzed the expression of chemokines and cytokines that induce lymphocytes, as well as the corresponding receptors for these molecules. The results revealed that certain chemokine ligands are highly expressed in SPP1+ TAM. In another study (138), SPP1+ TAMs were found to be enriched in the Wnt signaling pathway, which may support tumor growth (142–144).

### 4.5 Lipid-associated TAMs

LA-TAMs express characteristic genes involved in lipid metabolism, ECM degradation, and complement activation. Notable expressions associated with this subgroup encompass APOC1, APOE, C1QA, CCL18, MRC1, and MARCO. This subgroup is enriched in pathways related to lipid metabolism and oxidative phosphorylation (76), potentially exhibiting immunosuppressive functions that may facilitate tumor progression.

In a study on mucinous colorectal adenocarcinoma (MCA) (145), a population of MS4A4A+ TAMs characterized by high expression levels of C1QA and APOE was identified. Pseudotime analysis indicated that this group exhibited elevated gene expression during the later stages of tumorigenesis. Additionally, these cells express several markers associated with macrophage polarization, including MS4A4A, C1QB, C1QC, and SPP1. The authors also found that the expression of M2 macrophage marker genes in MS4A4A+ TAMs within tumor samples was significantly higher than that in normal tissues, suggesting a pro-tumor role for these cells in MCA progression. Previous studies have demonstrated that MS4A4A mediates M2 polarization of macrophages through activation of the PI3K/AKT and JAK/STAT6 pathways (146, 147). In the TCGA cohort, the authors validated that high expression levels of MS4A4A were correlated with poor prognosis. Experimental evidence further indicates that the proportion of MS4A4A+ TAMs is markedly enriched within the MCA TME, promoting immune suppression through interactions with specific tumor cells.

Another study (148) identified the specific presence of certain immunosuppressive cells in liver metastases, with a marked increase in SPP1+ TAMs and MRC1+ CCL18+ TAMs in colorectal cancer liver metastases. Notably, MRC1+ CCL18+ TAMs exhibit characteristics similar to Kupffer cells (149), suggesting a potential hepatic origin. Similar findings were observed by multiplex immunohistochemistry (mIHC). The authors also discovered that metastatic tumor cells tend to express the ligand CD47, an important “don’t-eat-me” signal (150), which may influence the anti-tumor immunity of MRC1+ CCL18+ macrophages via its corresponding receptor SIRPA (151). Differential expression of key molecules related to macrophage polarization was noted among MRC1+ CCL18+ TAMs; APOE exhibited anti-inflammatory properties and promoted M2 conversion (152), whereas MARCO (153) was significantly upregulated in liver metastasis-enriched MRC1+ CCL18+ macrophages. In contrast, we found that the enriched population of MRC1+ CCL18+ macrophages within CRC expressed higher levels of inflammatory cytokines, including TNF, IL1B, CCL3, and CCL4, indicating that even within the same subpopulation, distinct phenotypes may emerge in different tissues. A similar molecular profile shift was observed in SPP1+ macrophages, highlighting the functional changes shared by these immune cells at primary and metastatic tumor sites.

### 4.6 RTM-like TAMs

RTM-like TAMs refer to a group of macrophages that are characterized as being similar to normal RTMs. A study (85) identified two distinct functional states of macrophages in CRC: C1QC+ and SPP1+ macrophages. Specifically, C1QC+ macrophages exhibit phagocytic activity and antigen presentation capabilities, simultaneously, they may play a potential role in the process of recruiting or activating T cells. whereas SPP1+ macrophages are associated with promoting angiogenesis and facilitating tumor progression. Patients with high SPP1+ and low C1QC+ macrophage levels tend to have poorer prognoses. Through analyses such as RNA velocity, it has been demonstrated that C1QC+ TAMs are closely associated with a specific subset of RTMs and exhibit certain functional phenotypic similarities. Therefore, it is reasonable to propose that C1QC+ TAMs represent a type of RTM-like TAMs. Furthermore, only C1QC+ TAMs were identified in the colonic mucosa of individuals with ulcerative colitis and healthy subjects, whereas SPP1+ TAMs were not detected (154).

### 4.7 Proliferating TAMs

The research on proliferating TAMs is limited, primarily characterized by the expression of the proliferation marker MKI67. In the study conducted by Liu et al. (106), it was noted that MKI67+ TAMs exhibit similar marker genes to those previously characterized in C1QC+ TAMs; however, they uniquely express MKI67, which may be related to their proliferative function. Furthermore, RNA velocity analysis revealed a strong directional transition from MKI67+ TAMs toward C1QC+, further indicating that this population of TAMs possesses high proliferative capacity.

## 5 Discussion

CRC is one of the most prevalent malignant tumors worldwide, with an increasing annual incidence rate and a complex pathogenesis.

Based on the role of TAMs in cancer and their high plasticity, several therapeutic strategies targeting TAMs have been developed. For example, the reduction and depletion of TAMs are influenced by CSF1.Clinically, AMG 820, a fully human anti-CSF1 receptor antibody, inhibits the binding of its ligand CSF1 to interleukin 34 (IL-34), thereby preventing subsequent ligand-mediated receptor activation. This results in a decrease in the accumulation of immunosuppressive TAMs in solid tumors (155). Chemokine ligand 2 (CCL2) recruits monocytes expressing chemokine receptor 2 (CCR2) from the peripheral blood to the tumor site, where they further mature into TAMs (156). Therapeutic blockade of the CCL2/CCR2 axis suppresses the recruitment, infiltration, and M2 polarization of inflammatory monocytes mediated by TAMs. This leads to the reversal of immune suppression within the TME and activates anti-tumor CD8+ T cell responses (157). Clodronate is a chemical agent that induces macrophage depletion and significantly reduces TAMs in the TME, thereby diminishing their pro-angiogenic effects (158). The triggering receptor expressed on myeloid cell 2 (TREM2) represents a potential therapeutic target for modulating immunosuppressive TAMs (159). The use of TREM2 inhibitors and similar tools selectively eliminates immunosuppressive macrophages, restores normal immune responses, and inhibits tumor growth (160). Additionally, TAMs can be repolarized into M1-like macrophages to regain their anti-tumor properties. Toll-like receptors (TLRs) are crucial pathogen-recognition receptors expressed by immune cells. The utilization of TLR agonists is an effective strategy for rapidly activating both innate and adaptive immunity (161). Following TLR-3 stimulation, the upregulation of M1-specific markers, such as major histocompatibility complex class II molecules (MHC II), and co-stimulatory molecules, such as CD86, CD80, and CD40, is observed in M2 macrophages. In contrast, there is a decrease in the expression of M2 markers, including CD206, Tim-3, and proinflammatory cytokines. This phenotypic shift effectively inhibits tumor growth (162). In addition, TAMs exhibit anti-tumor properties that can be leveraged. For instance, using SIRP1α-CD47 inhibitors and anti-MS4A4A antibodies, certain unique TAM subpopulations can be utilized to impede tumor growth or restore T cell-mediated anti-tumor immunity (147, 163). Furthermore, TAMs express PD-1, and the expression level of PD-1 is negatively correlated with the phagocytic capacity of macrophages. Blocking the PD-1/PD-L1 pathway can enhance macrophage-mediated phagocytosis of tumor cells and inhibit tumor progression (164). Macrophages are critical components of the complex interplay between the immune system and tumors; thus, they represent significant therapeutic targets for cancer prevention and treatment. In response to tumor stimuli, TAMs undergo M2-like polarization, promoting tumor growth and severely affecting prognosis. Therefore, the development and application of drugs based on TAMs and the TME are of substantial significance (36).

The article discusses several specific subpopulations of tumor-associated macrophages (TAMs) that have been the focus of various treatment-related studies. One study (165) demonstrated that the knockout of SPP1 significantly inhibited the infiltration of M2 TAMs in xenograft tumors, while RNA aptamer-based blockade of SPP1 markedly suppressed tumor growth and M2 TAM infiltration in mouse models. Additionally, literature reports (166)indicate that a certain compound can downregulate SPP1 both *in vitro* and *in vivo*, leading to tumor remission in different mouse models of cancer. Research by Zhang et al. (167) demonstrated that targeting C1Q+ TAMs effectively alleviates immune suppression and enhances the efficacy of immune checkpoint blockade therapy. These findings suggest that specific subpopulations of macrophages may serve as potential targets for therapeutic intervention, thereby presenting new opportunities for cancer treatment.

TAMs, which differentiate from monocytes, play a crucial role in tumor immunity in CRC. The M1/M2 polarization subtypes provide a foundational framework for summarizing certain functions of macrophages; however, an increasing body of research (94, 168) has demonstrated the complexity of TAM functions and phenotypes. The advent of technologies such as single-cell RNA sequencing (scRNA-seq) has further substantiated these findings at a more precise level, emphasizing the necessity of leveraging new techniques to enhance existing molecular identification systems based on previous knowledge. However, given the limited number of single-cell studies focusing on specific TAM subpopulations, the relationships among these unique subtypes still require further investigation. Additionally, some TAM subtypes identified in previous studies are challenging to classify within the existing functional subsets based on their characteristics, transcription factors, enriched pathways, and predicted functions. For instance, in a study by Bao et al. (169), a distinct population of S100A9+ macrophages was identified, leading to an immunosuppressive phenotype cluster described as immune tolerance in CRC at the single-cell level. However, additional information is needed for a more accurate characterization of this subgroup. In another study integrating CRC tissues with adjacent normal tissues (170), seven specific macrophage subpopulations were identified. Among these, IDO1+ TAMs were the only macrophage subtype predominantly present in CRC tissues. It has been observed that IDO1+ macrophages interact with various cell types; however, a clear understanding of these interactions is still lacking. Several large-scale pan-cancer studies utilizing single-cell analyses (171, 172) have identified unique subpopulations. For example, MMP9+ TAMs may play a role in tumor tissue remodeling (173–175), CX3CR1+ TAMs may exhibit unique phagocytic patterns (176, 177), ECM-associated TAMs (ECMMac) involved in extracellular matrix remodeling are upregulated (178, 179), and SPP1AREGMac exhibit distinct distribution within tumor tissues. This evidence further underscores that the polarization of macrophages into M1 and M2 phenotypes is not the sole critical factor in cancer. Therefore, the functional diversity of macrophages should be comprehensively considered. With sufficient research, it may become possible to establish a novel molecular classification system for CRC based on TAM functionality. Such an approach would allow for a more detailed characterization of TAM heterogeneity, thereby facilitating the development of targeted therapies for specific TAM populations in CRC.
